# Gap Formation at Luting Interfaces of CAD/CAM Ceramic and Composite Partial Crowns Assessed by OCT

**DOI:** 10.3390/dj14020116

**Published:** 2026-02-17

**Authors:** Nadia Oberück, Dennis Palsa, Tobias Meißner, Marco Pellino, Rainer Haak, Ellen Schulz-Kornas, Dirk Ziebolz

**Affiliations:** 1 Department of Cariology, Endodontology and Periodontology, Leipzig University, Liebigstraße 12, 04103 Leipzig, Germany; dennis.palsa@medizin.uni-leipzig.de (D.P.); tobias.meissner@medizin.uni-leipzig.de (T.M.); rainer.haak@medizin.uni-leipzig.de (R.H.); ellen.schulz-kornas@medizin.uni-leipzig.de (E.S.-K.); 2 Department of Conservative Dentistry and Periodontology, Brandenburg Medical School Theodor Fontane (MHB), Wilhelmsdorfer Str. 57, 14776 Brandenburg an der Havel, Germany; dirk.ziebolz@medizin.uni-leipzig.de; 3 Institute for General Practice, Faculty of Medicine, Philipp-Rosenthal-Straße 55, Haus W, 04103 Leipzig, Germany

**Keywords:** CAD/CAM, ceramics, resin-based composites, interface, OCT, TCML

## Abstract

(1) **Background/Objectives**: Gap formation contributes to the clinical failure of partial crowns. Therefore, it was analyzed at the interfaces between restoration, luting material, and tooth in partial crowns made of lithium disilicate ceramic (LS2) and nanohybrid composite (RBC) after thermomechanical loading (TCML) using optical coherence tomography (OCT). (2) **Materials and Methods:** Sixteen human mandibular molars were restored with CAD/CAM partial crowns made of LS2 (IPS e.max^®^ CAD) or RBC (Tetric^®^ CAD) using adhesive cementation (Variolink^®^ Esthetic DC). The restorations were imaged by OCT (1550 nm, 28 kHz) at t0 = 24 h, t1 = 90 days of water, t2 = after TCML with 480,000 loading cycles, and t3 = TCML with 1,200,000 loading cycles. Gap lengths (%) at interface 1 (partial crown-luting material) and interface 2 (luting material–enamel/dentin) were quantified. Groupwise and pairwise comparison of OCT parameters was conducted using the Mann–Whitney U, Friedman, and Conover–Iman tests with Bonferroni correction (α = 0.05). (3) **Results:** At interface 1, LS2 showed a larger median gap length than RBC (ceramic = 48.4%; composite = 5.2%, *p* < 0.01). At interface 2, the largest median gap length for LS2 was measured at the dentin (ceramic = 59.7%; composite = 52.5%), while for RBC, the enamel was more affected (ceramic = 26.2%; composite = 36.9%). (4) **Conclusions:** OCT enables reliable gap detection in partial crowns under functional loading and is therefore suitable for monitoring adhesive interface integrity. Under in vitro conditions, both materials demonstrated stable adhesive performance without debonding, while material-dependent differences in gap formation and distribution were observed.

## 1. Introduction

The increasing demand for esthetic, minimally invasive, yet functional restorations has led to further development of indirect restorative materials in modern dentistry [1]. As a result, defect- and material-oriented preparation designs play an ever more important role in direct and indirect tooth-colored restorations [2]. In cases of extensive single-tooth defects in the posterior region, covering the cusps for fracture protection with indirect crowns or partial crowns may be a more appropriate treatment approach than a direct restoration [3,4,5]. In particular, partial crowns can be used for this purpose and can be designed to be more defect-oriented and less invasive [6]. Furthermore, these restorations can be produced using CAD/CAM technology, a method established in dentistry since the 1980s [7]. In addition to time and cost efficiency, an advantage of this procedure is the improved structural quality of the CAD/CAM block materials resulting from the manufacturing process [8,9]. In order to cover a wide range of indications, an increasing number of CAD/CAM materials are available for use in daily practice [9,10,11]. These ceramics can be classified based on their microstructure into glass-ceramics, polycrystalline ceramics, and resin-matrix (hybrid) ceramics [12], reflecting differences in processing behavior, mechanical properties, and clinical indications [13]. Among glass ceramics, lithium disilicate ceramic (LS2) is widely used for single-tooth restorations, such as partial crowns, as it combines high translucency alongside sufficient strength required for mastication [14]. Nowadays, partial crowns can be made from established ceramics and new materials like resin-based composites (RBCs) [15,16,17]. The advantages of RBC materials are cost reduction and shortened manufacturing time, as necessary steps and materials in the manufacturing process are omitted in comparison to ceramic materials [18,19]. However, the available data comparing LS2 and RBC materials are heterogeneous. While some in vitro studies pointed out disadvantages of composites over well-established ceramics [15,17,20], other in vitro studies showed beneficial characteristics of CAD/CAM composites [21,22,23]. Thus, these materials can be considered alternative restorative materials [21,22,23]. First clinical data of a 5-year follow-up study showed survival and success rates of 96% and 85.7–100% for indirect inlay, onlay, and overlay composite restorations and 97% and 93.3–100% regarding corresponding ceramic restorations [24,25]. In contrast, 10- and 15-year follow-up data are available for ceramics only, including partial coverage restorations, and achieved similar survival rates compared to complete coverage restorations [6]. The few long-term clinical studies are probably too heterogeneous in their study design and do not indicate a clear trend, yet they reveal initial causes of failure.

Reasons for failure of CAD/CAM restorations include material or tooth fractures [21,26,27], as well as the quality of the adhesive bond at the restorations’ interfaces and margins [3,7,11,27]. Gap formation, caused by stress or insufficient luting procedures, can lead to caries lesions at the restoration margin and/or partial or complete debonding, i.e., complete loss of the restoration [3,7,21,25,26,27]. CAD/CAM materials exhibit substantial differences in elastic modulus and flexural strength, both of which are expected to influence deformation behavior, stress distribution, and stress transfer within the adhesive complex under functional loading (Table 1). Such material-dependent mechanical properties govern interfacial stress development and may thereby contribute to differences in gap formation at the interfaces between the restoration, luting material, and tooth substrate [11,27]. It is thus important to detect changes at the interfaces early in the wear process of the restoration. Clinical monitoring usually includes X-rays to image and assess the restoration quality. The main disadvantages of conventional X-rays are, on the one hand, the exposure and invasiveness, and, on the other hand, the difficult and sometimes inadequate assessment of the restoration margins if the relevant areas are not projected without overlapping [28].

Optical coherence tomography (OCT) is a non-invasive technique that allows more precise information to be obtained regarding material defects, interfacial adhesive defects, and structural defects of the adjacent enamel and dentin, since the restorations do not have to be destroyed, and changes over time can be visualized [29,30]. In this context, OCT enables the visualization of early interfacial changes, while the interpretation of the underlying failure mechanisms must be derived from material-related mechanical considerations. Yet, existing data on OCT focuses mainly on direct adhesive restorations and the detection of carious lesions, while knowledge regarding indirect restorations remains limited.

This study aimed to analyze the interfacial bonding of partial crowns with two different CAD/CAM materials (ceramic and composite) before and after thermo-mechanical loading (TCML) using SD-OCT.

(1) OCT was hypothesized to be a suitable method for interface visualization and defect detection in partial crown restorations.
(2) Furthermore, it was hypothesized that LS2 would show fewer gaps than RBC at the two interfaces between restoration and luting material (interface 1) (2a) and luting material and the tooth substrates, enamel and dentin (interface 2) (2b).

## 2. Materials and Methods

### 2.1. Material and Specimen Preparation

**Figure 1:**
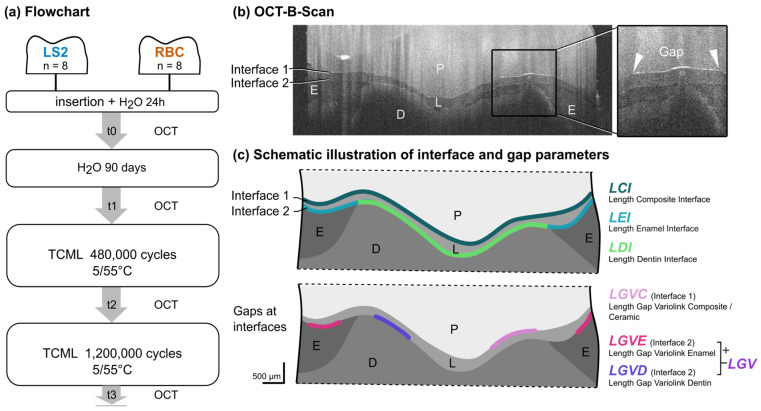
Flowchart of the experimental design (**a**), selected OCT B-scan (**b**), and schematic illustrations of seven interface and gap parameters (**c**). Interfaces in pixel and outcome parameters expressed as % gap; RBC = resin-based composite, LS2 = lithium disilicate ceramic, TCML = thermo-mechanical loading, P = partial crown, L = luting material, E = enamel, D = dentin.

For this in vitro study, 16 visually caries-free extracted human lower molars were collected after written informed consent had been obtained from all patients using a standardized form and stored in 0.5% chloramine T solution at 4 °C immediately after extraction for no longer than six months before use. The criteria for inclusion were complete root growth and an untreated root canal system. All teeth were randomly assigned into two groups (*n* = 8) based on the CAD-CAM material that was used: lithium disilicate ceramic (LS2 group) or resin-based composite (RBC group; Figure 1, Table 1). The randomization, including allocation concealment, was conducted to avoid preselection based on tooth morphology and avoid human bias (for R code, see Supplementary Materials file S1). The sample size was determined a priori by a simulation-based power analysis, which indicated that eight samples per group were sufficient to detect small-to-medium effect sizes (Wilcoxon effect size *r*; see Supplementary Material file S2 for details).

Specimen restoration was performed by a calibrated dentist (trained in standardized preparation, CAD/CAM procedure, and adhesive luting) using loupe glasses (magnification of 2.8) and special diamond burs adjusted for the fabrication of partial crowns (expert-set ceramic inlays and partial crowns 4562, Komet Dental, Lemgo, Germany).

In order to provide a standardized preparation, only mandibular molars with comparable morphology and dimensions were included in the study, and tooth preparation was conducted following the guidelines for indirect ceramic restorations [2]. All tooth cusps were included and uniformly reduced by 2 mm, with buccal and lingual preparation margins positioned above the anatomic tooth equator, resulting in complete cuspal coverage.

Proximal reduction was set to 4 mm in the mesial and distal boxes, while maintaining preparation margins above the enamel–cement junction. The central cavity floor was prepared to a depth of 2 mm. All preparations were performed using depth-marked burs (expert-set ceramic inlays and partial crowns 4562, Komet Dental, Lemgo, Germany) to ensure reproducibility. Visual inspection was carried out to confirm dentin exposure at the central cavity floor.

As part of quality assurance, each preparation was evaluated by an experienced, calibrated dentist, and minor adjustments were made when necessary to ensure consistency across all specimens.

### 2.2. Restorative Procedure

The specimens were digitally scanned (CEREC Primescan, Cerec 5, Dentsply Sirona, York, PA, USA). To improve light penetration during SD-OCT measurements, the restorations were manually designed (inLab CAM, version 20.0.1.203841, Sirona Dental Systems, Bensheim, Germany) with a reduced anatomic structure and a maximum occlusal layer thickness of 3.5 mm in cusp areas.

Restorations were milled with a minimum occlusal layer thickness of 1.5 mm and a luting space of 80 µm (CEREC MC XL, Sirona Dental Systems, Bensheim, Germany) using block size C14 (A3/HT). Before insertion, all restorations were carefully inspected under magnification to detect potential defects, including fractures, chipping, surface contamination, or excess glaze on interfacial areas. Restorations presenting any of these imperfections were excluded or corrected prior to placement. The partial crowns were then pretreated and inserted adhesively according to the manufacturer’s recommendations (Table 1). Tooth surfaces were conditioned with phosphoric acid (30 s enamel, 15 s dentin, etch-and-rinse application, 37% phosphoric acid, Ivoclar Vivadent, Schaan, Liechtenstein) and single-component, light-cured adhesive (Adhese Universal VivaPen, Ivoclar Vivadent, Schaan, Liechtenstein).

### 2.3. Artificial Aging

After restoration, the specimens were stored for 90 days in deionized water at 37 °C in a heat cabinet (WTB binder MB6, 7200 Tuttlingen, Germany). Subsequently, thermo-mechanical loading (TCML, chewing simulator CS 4.8, SD Mechatronics, Feldkirchen-Westerham, Germany) was performed. All specimens were coated with a 1 mm thick layer of polyether material (Impregum, 3M Espe, Seefeld, Germany) to simulate the human periodontal ligament and embedded in a cold polymerizing resin (Technovit 4000, Kulzer, Wehrheim, Germany). To ensure a homogeneous layer thickness of polyether, each specimen was first immersed in wax heated to 70 °C and subsequently allowed to cool (Wapfeno-modeling wax, VEB Wachswarenfabrik, Nordhausen, Germany). Before embedding, excess wax accumulations surrounding the roots were carefully removed, and the remaining wax layer was manually adjusted and verified with a periodontal probe to ensure a uniform thickness around the root surface. Subsequently, the specimens were embedded in Technovit. After polymerization, the wax was removed from the embedding mold and replaced with Impregum. Specimens were positioned in the holders using a 3D-printed fixture (Objet30 Dental Prime, Stratasys, Rheinmünster, Germany) to maintain standardized centric load transfer. Unintentional movements during embedding and resultant deviations in the functional load axis were avoided. To simulate an approximate five-year in situ performance, intermittent TCML (5 × 600 thermocycling, 5 °C/55 °C, deionized water, mechanical loading of 1.2 × 10^6^ with a load of 50 N per specimen) was performed. The vertical and horizontal speed was set to 6 mm/s (1.6 Hz), resulting in a forward-backward movement of 0.5 mm while contacting the sample. Steatite balls (CeramTec, Plochingen, Germany) with a diameter of 6 mm were used as standardized antagonists to carry out a physiological motion of axial mastication. When thermocycling was completed, the specimen holders remained filled with deionized water (30 °C) until mechanical loading was finished.

### 2.4. OCT Imaging and Outcome Parameter

The restorations were imaged by SD-OCT (Telesto-II Sp21, 1550 nm, 28 kHz ThorImage OCT version 5.4.2.0; Thorlabs, Dachau, Germany). All samples were examined at four points in time: baseline = t0, after water storage (t1), after TCML at 480,000 (t2), and 1,200,000 cycles (t3; Figure 1a). The examiner was trained by an experienced analyst (12 years of OCT imaging) on one data set. Ten data sets were used for intra- and inter-personal calibration (repeated reads) with a standard deviation of <5% between the experienced analyst and the examiner. The analyses were blinded for specimen numbers and time points.

**Figure 2:**
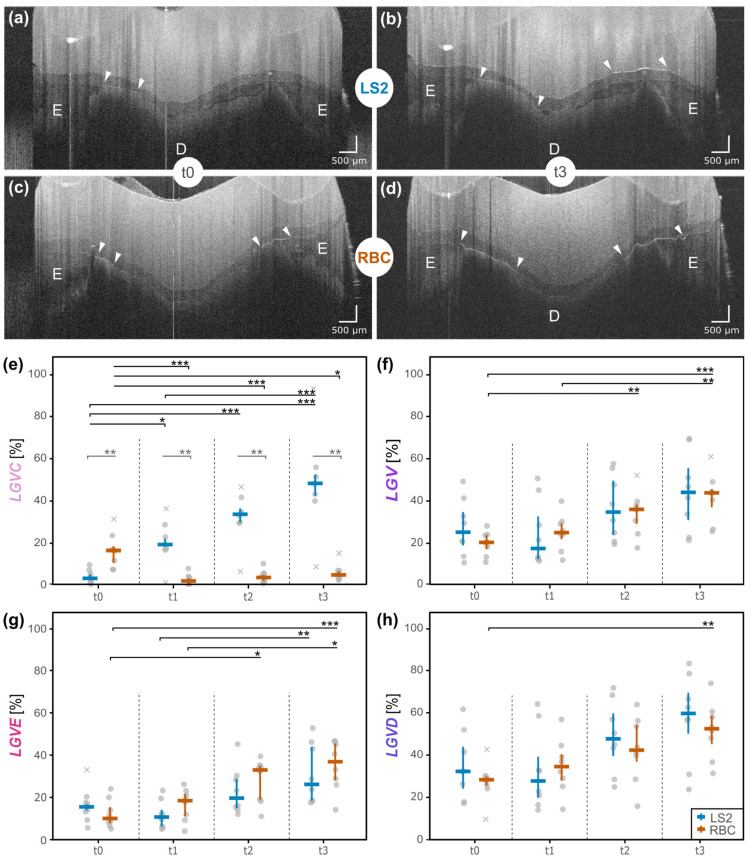
Selected OCT B-scans of LS2 = lithium disilicate ceramic partial crowns (**a**,**b**), RBC = resin-based composite (**c**,**d**). Gap formations (white arrows) are found in the tooth tissues, enamel (E) and dentin (D). Strip charts (**e**–**h**) of the OCT parameters (Figure 1) are given at four steps in time (after 24 h water storage (t0), 90 days water storage (t1), after 480,000 thermo- and mechanical loading cycles (t2), and 1,200,000 cycles (t3)); parameter names: LGVC = length gap Variolink composite/ceramic, LGVE = length gap Variolink enamel, LGVD = length gap Variolink dentin, LGV = length gap Variolink; grey circle = individual measurement, grey x = outlier, significance levels: * *p* < 0.05, ** *p* < 0.01, *** *p* < 0.001.

The technical specifications of the SD-OCT were as follows: sensitivity ≤ 106 dB; power on sample 4.5 mW; spot size 22.5 µm; bandwidth = 230 nm; field of view (max.) 16 mm × 16 mm; axial resolution (air) ≤ 7.3/5.5 µm; A-scan averaging 1, voxel size x/y/z = 10/20/5.41 µm. To enable ideal and reproducible positioning of the specimens while imaging occlusal surfaces, a 3D-printed fixture (Objet30 Dental Prime, Stratasys, Rheinmünster, Germany) was used. The specimens were fixed at an angle of 90 degrees so that the measurements could be taken face-on to the specimen. Twenty-five equidistant OCT B-scans per restoration were extracted [29] from the complete volume scan, with the first and last OCT B-scans defined by the start and end of the partial crown (Figure 2a–d). The OCT B-scans were analyzed via customized routines in Image J (version 1.52p, open-source image processing and analysis in Java, Wayne Rasband, National Institutes of Health, Bethesda, MD, USA), following a standardized protocol in which interfaces were manually traced using a custom polyline-based tool. The examiner placed sequential points along the structure of interest (interfaces and gaps), generating a set of vertices converted into smooth curves via spline fitting.

The quantitative evaluation of the images was conducted using the following continuous outcome parameters: interface 1 (restoration to luting agent (LCI)) and interface 2 (luting agent to either enamel (LEI) or dentin (LDI)). Gap lengths of the respective interfaces were measured (LGVC, LGVE, LGVD, LGV (LGVE + LGVD; in pixels, gap parameters expressed as % gap, for description see Figure 1b,c). Subsequently, the weighted mean gap length was calculated as the relation as follows:

$$Mean\;Gap\;\left(\%\right)=\;\frac{\sum_{i=1}^{n}g_{i}}{\sum_{i=1}^{n}L_{i}}\times 100$$

where $g_{i}$ is the gap length (in pixels), $L_{i}$ is the interface length (in pixels), and n is the number of OCT B-scans.

### 2.5. Statistical Analysis

The open-source software R (Windows, R-4.4.1, R Development Core Team 2024) was used for data processing and statistical analyses. The R packages are given in the Supplementary Information (file S1). Data exploration of determined parameters was conducted by using means, medians, interquartile ranges, and strip charts. The data was inspected by checking normality assumptions using residual plots and the Shapiro–Wilk test; Levene’s test was applied to assess for homogeneity of variances. Due to small sample sizes and partly non-normally distributed data, we used the following non-parametric tests: Mann–Whitney U test for groupwise comparison (material at each time point), Friedman test for within-group comparison (time points), and post hoc pairwise Conover–Iman test. For all tests, the significance level was set to α = 0.05 using Bonferroni correction.

## 3. Results

The survival rate of the restorations after water storage and TCML was 100%, with no debonding observed. There were no chippings, cracks, or any visible fractures to the samples. Both materials, RBC and LS2, showed an increase in the OCT signal at the interfaces, indicating a gap (white lines marked with triangles) after artificial aging (t3) (Figure 2a–d, Supplement file S1).

At interface 1 (LCI; restoration to luting composite), LS2 showed a highly significant continuous increase in gap formation (LGVC) from t0 to t2/t3 (*p* < 0.001, effect size = 0.9, Figure 2e, Supplement file S1 Tab.9). In contrast, for RBC, there was a decrease after 90 days (t1) (*p* < 0.001, effect size = 0.9) up to 1,200,000 of TCML cycles (t3) (*p* < 0.05, effect size = 0.9) with a subsequent, but very minor, increase after TCML started. LS2 displayed the most extended gaps over time (t3: LGVC median LS2 = 48.4%; LGVC median RBC = 5.2%, *p* < 0.001, effect size 0.8, Figure 2, Supplement file S1 Tab.7). In addition, no cracks or fractures were found at interface 1 or in either of the restoration materials.

At interface 2 (luting composite to tooth substrate), the parameter values (LGV/LGVE/LGVD) in both materials exhibited a continuous gap increase over time (Figure 2f–h, Supplement file S1 Tab.9). LS2 showed higher gap values in dentin (t3: LGVD median LS2 = 59.7%; LGVD median RBC = 52.5%; Figure 2h), whereas RBC showed them in enamel (t3: LGVE median LS2 = 26.2%; LGVE median RBC = 36.9%, Table 2, Figure 2g). Nevertheless, the overall longer gaps were found in dentin for both materials (Figure 2h).

## 4. Discussion

This in vitro study identified material-dependent differences in measurable gap formations and associated adhesive failures at the interfaces with the tooth substrate and restoration material. After TCML, partial crowns made of LS2 showed significantly more gap formation between restoration and luting material (interface 1) than partial crowns made of RBC. However, gap formation between luting material and enamel/dentin (interface 2) was similar for both materials (Figure 2f). Overall, a higher percentage of gaps was measured at the dentin surface than at the enamel.

In the literature, LS2 is often described as superior to RBC over an observation period of five years, but this mainly refers to crowns or inlays [25]. Complete debonding is discussed as a frequent cause of loss in RBCs, while major irreparable chipping is a cause of failure in ceramics [31,32,33]. Nevertheless, other clinical studies demonstrated a similar clinical performance of CAD/CAM partial coverage restorations made of LS2 or RBC after a one- or two-year follow-up [34,35,36]. However, the focus of these studies was on the restoration margin. No significant differences were found in marginal adaptation, discoloration, or secondary caries [34]. The only interfacial criterion considered by Hassan et al. was debonding, which only occurred within the ceramic group [34]. Currently, no studies are available comparing the quality of the adhesive bonding at these two materials’ interfaces (LS2 vs. RBC), either when used as partial crowns or with particular reference to their different mechanical properties (e.g., Young’s modulus, E). However, finite element analyses (FEA) have investigated the stress distribution of ceramic and composite crowns after loading, considering their E-moduli [11,37]. It is assumed that combining the materials with E-moduli comparable to those of tooth tissues leads to force transmission between materials, while significant differences in E-moduli may result in a force accumulation within the material [37]. Thus, FEAs indicate that the highest stresses were measured for ceramics within the restoration and for composites within the tooth structures [11]. In a study by Yazigi et al., which focused on thin occlusal LS2 veneers, similar results were obtained, with cracks occurring in the ceramic restoration, while the luting material and underlying tooth structures showed no alterations [38]. Similar observations have been made regarding the present in vitro study, which depicted the stress distribution in terms of visible and, thus, measurable gap formations. It is assumed that stresses in the LS2 ceramic appeared to be transmitted to the adjoining interface (interface 1) and are not accumulated in the restoration itself (Figure 2d).

In addition to the elastic modulus, flexural strength may be considered a further mechanical parameter influencing the observed interfacial behavior [39,40]. LS2 ceramics generally exhibit higher flexural strength values than RBC (530 MPa vs. 272 MPa, Table 1). The comparatively high flexural strength of LS2 may enable the restoration to withstand bending-induced stresses without macroscopic fracture while simultaneously promoting stress transfer towards the restoration–luting material interface (interface 1). This mechanism could explain the increased gap formation observed at this interface, despite the absence of visible cracks or fractures within the LS2 ceramic. The lack of structural damage within the restoration itself may therefore be interpreted as a consequence of the high intrinsic strength of LS2, with stresses being preferentially accommodated at the adjoining adhesive interface rather than within the ceramic material. In contrast, the lower flexural strength and increased deformability of RBC may allow for partial stress dissipation within the restoration material, potentially resulting in a greater transmission of stresses towards the tooth–luting material interface (interface 2). Consequently, due to the higher gap values measured for the LS2 ceramic at interface 1 (LGVC), the initial hypothesis (a), assuming generally lower gap values for LS2 ceramics, must be rejected.

Comparing the present results to clinical settings, it is repeatedly observed that after debonding of ceramic partial crowns, luting materials remain adherent to the tooth structures [21]. This observation would be consistent with the current findings that the partial crowns made of RBCs do not have significantly higher measured gap values at interface 2 (LGV) than LS2 ceramics (hypothesis b). It is to be assumed that the forces are further transmitted to the underlying tooth structures. Thus, the hypothesis for interface 2 has to be partly rejected. However, there was a trend for the RBCs to exhibit a more expansive signal line, indicating a larger gap expression. As this only affected the thickness of the gaps, it had no influence on the data. It is hypothesized that if the specimens had been loaded longer, there would probably have been increasing failure with increasing time and more significant differences between the two materials. In future studies, it is therefore advisable to consider both the length of the gaps and the extent of the signal line as a criterion.

Looking at the gap development at interface 2 regarding enamel and dentin (LGVE/LGVD), the overall gap formation on dentin was more significant than on enamel (Figure 2g,h), confirming previous findings that there is a better bond to enamel [41]. Regarding the different indirect materials, RBC showed a trend towards larger gap values at the enamel (LGVE) compared to LS2. Due to a large difference in the E-moduli (enamel: 86 GPa, RBC: 10 GPa), one would expect the opposite result (hypothesis b). Compared to the LS2 ceramics, however, RBCs are prone to water absorption (Table 1), which could lead to a gap progression in marginal areas and, thus, at the enamel [42]. The water absorption causes swelling of the composite, causing pumping effects and a centrally directed gap increase [18].

Although a 90-day water storage period was included prior to TCML in order to induce initial hydrolytic effects, short-term storage in deionized water cannot fully reproduce the complex biochemical and enzymatic degradation mechanisms of the oral environment. Hydrolytic degradation may therefore further evolve over longer aging periods or in more aggressive media. This limitation may have influenced the observed material-dependent differences, as RBCs are more susceptible to hydrolytic degradation than lithium disilicate ceramics, potentially affecting gap formation and progression.

While in the current study, a thermo-mechanical loading of five years is simulated using TC (5/55 °C), the actual oral residence time is not considered separately. Total water storage equivalent to intraoral exposure was about four months (Figure 1a). Even though the temperature varied between 5 and 55 °C during the chewing simulation, and the number of chewing cycles was taken as an approximation of 5 years in vivo following previous studies (see Heintze et al.), there are experimental limitations [43]. The experimental settings only partly reflect the in vivo situation and an adequate duration of exposure to human saliva. In this in vitro study, TCML was chosen to simulate the loading of the partial crowns, as this in vitro method can mimic the dynamic loading similar to the chewing mechanism and, at the same time, mimic the moist environment of the oral cavity. Rosentritt et al. have already demonstrated that clinical use can be simulated with TCML settings of 1,200,000 × 50 N and 6000 thermal cycles [44]. These settings were also selected in this study, as they are considered equivalent to a clinical wearing time of 5 years [44]. However, the rigid fixation, uniform movement, and consistent loading are disadvantages of the TCML method, as it disregards individual chewing patterns [33]. In order to minimize these shortcomings and to simulate the natural mobility of the teeth through the periodontium, in the present study, the roots of the specimens were coated with a 1 mm layer of the polyether material Impregum™ as established in previous studies [18,44].

In addition, tooth preparation was performed manually by an experienced operator rather than by a fully standardized mechanical protocol. This approach allowed individual adaptation to tooth anatomy and preservation of substrate-specific characteristics, particularly with respect to enamel and dentin distribution. At the same time, manual preparation is inherently operator-dependent and may introduce a degree of variability despite careful verification and calibration.

Various methods are described in the literature to detect changes within the area of restorations, luting materials, and tooth substances, as well as their interfaces, and to determine reasons for failure. Common test methods include radiography, microleakage analysis, micro-computed tomography, and scanning electron microscopy [35,36,45]. However, several of these approaches are either invasive, associated with ionizing radiation, or limited with regard to longitudinal assessment under clinically translatable conditions [45].

In contrast, the OCT method offers non-invasive, real-time imaging with high axial resolution and a pronounced sensitivity to early optical discontinuities at adhesive interfaces [38,46]. This enables the detection of incipient interfacial changes before macroscopic failure or debonding becomes clinically evident. Importantly, OCT provides a clear pathway towards clinical translation, as it can be applied without radiation exposure and with potential for chairside monitoring.

A methodical limitation of OCT is its material- and thickness-dependent penetration depth, which may restrict image acquisition in restorations with higher layer thicknesses or in areas beneath cusps. This aspect was considered in the present experimental setup by limiting occlusal thickness to facilitate sufficient light penetration. Against this background, OCT and micro-computed tomography should be regarded as complementary rather than interchangeable techniques [47,48], with OCT being particularly suited for longitudinal monitoring of adhesive interface integrity under clinically relevant conditions.

Several studies have been published in dentistry using SD-OCT to examine carious lesions or composite restorations [29,49]. The field of indications for OCT has been expanded in recent years to include indirect materials like veneers and partial crowns [10,38,46].

Clinical studies are considered particularly valuable in assessing the clinical prognosis of restorations. However, in addition to patient adherence, which must be maintained for as long as possible, one difficulty is that detailed analyses of individual sub-parameters of clinical success are complex. These issues can often be addressed with in vitro studies. Teeth, restorations, and luting materials can be viewed from all sides and analyzed using various techniques, such as OCT [50]. Even though this in vitro study showed structural changes in the interfacial areas, all restorations were still in situ after TCML. OCT is a method of visualizing early interface failure and can be used prospectively to prevent more severe complications such as total debonding or fracture. A methodological limitation is the limited and varying penetration depth of OCT. A study by Challakh et al. showed that SD-OCT penetration depth is material-dependent. Zirconia-reinforced lithium silicate ceramics, lithium aluminum silicate glass ceramics, and LS2 could be examined very well by OCT with penetration depths of 4 mm and more, whereas zirconia, polymer-infiltrated, and feldspar ceramics could hardly be penetrated by OCT due to their low light transmission (<1 mm) [10]. Especially in restorations with high layer thickness or in areas under cusps, the diagnostics could thus be a limiting factor for this examination method. This clinically relevant, thickness-dependent attenuation of transmitted light through lithium disilicate has also been demonstrated in polymerization studies, where increasing ceramic thickness and darker shades markedly reduce the energy reaching the luting material and require extended exposure times to achieve acceptable conversion [51]. Another limiting factor is that the evaluation of OCT images relies on the examiner’s expertise and consistent inter-examiner calibration, as no software-based analysis tools are currently available.

Further studies should be conducted, particularly in indirect restorations, to validate the OCT method for wider application in clinical settings. In addition, since the focus of the current in vitro study was on interfacial performance, examinations of changes within the tooth structures and indirect materials, such as (in)fractures or material inhomogeneities, as well as the restoration margin, would be desirable.

In future studies, the applied water storage and TCML protocols could be extended to simulate longer clinical wear and include larger sample sizes to improve statistical robustness. Modified loading conditions with increased or variable forces, as well as mechanical testing until failure, may further enhance the understanding of the relationship between gap formation, interfacial degradation, and overall restoration performance.

In addition, the inclusion of a broader range of indirect CAD/CAM materials and a variation in restoration thicknesses may provide further insight into material- and geometry-dependent limitations. Experimental evaluation under thickness conditions approaching or slightly below manufacturer recommendations could help to explore more critical boundary conditions relevant to clinical practice.

## 5. Conclusions

Within the limitations of this in vitro study, the following conclusions can be drawn:

1. Gap formation in adhesively luted LS2 and RBC partial crowns can be detected by SD-OCT.
2. The extent and distribution of the gaps are material-dependent, both at the interface of the luting composite to the restorative material and at the enamel and dentin.
3. From a clinical perspective, both materials maintained stable retention throughout the investigated loading period despite the presence of interfacial gaps. Microscopically detectable gap formation, therefore, does not necessarily indicate immediate clinical failure but may represent potential sites of interfacial weakening over time. In this context, OCT may support the clinical monitoring of adhesive interfaces by visualizing early interfacial changes while restorations remain in situ.

## Figures and Tables

**Table 1:** Composition, mechanical properties, and pretreatment of the investigated CAD/CAM materials according to the manufacturer’s recommendation.

Code	Classification, Product Name	Minimal Material Thickness (mm)	Pretreatment, Luting Material	Composition (wt%)		Water Sorption (µg/mm^3^)	Density (g/cm^3^)	E-Modulus (GPa)	Flexural Strength (MPa)
LS2	Lithium disilicate ceramic IPS e.max^®^ CAD ^a^	1.0	IPS^®^ Ceramic Etching Gel ^a^ Monobond^®^ Plus Universal Primer ^a^ Variolink^®^ Esthetic DC ^a^	SiO_2_	57–80	0	2.50 ± 0.10	95	530
				Li_2_O	11–19				
				K_2_O	0–3				
				P_2_O_5_	0–1				
				ZrO_2_	0–8				
				ZnO	0–8				
				Al_2_O_3_	0–5				
				MgO	0–5				
				Coloring oxides	0–8				
				UDMA, TEGDMA	/				
RBC	Resin-based composite Tetric^®^ CAD ^a^	1.5	Al_2_O_3_ (50 µm, 1, 5 bar) Adhese^®^ Universal ^a^ Variolink^®^ Esthetic DC ^a^	Nanohybrid filler	86	22.5	n/s	10	272
				UDMA + DMA	14				

UDMA = Urethandimethacrylat; TEGDMA = Triethylenglycoldimethacrylat; DMA = Dimethacrylat; n/s = not specified. ^a^ Ivoclar Vivadent (Schaan, Liechtenstein).

**Table 2:** Results of the % gap length. OCT-parameters displayed by groups and time steps (t0–t3); medians [interquartile range] were calculated; RBC = resin-based composite (a, b), LS2 = lithium disilicate ceramic, LGVC = length gap Variolink composite/ceramic [%], LGVE = length gap Variolink enamel [%], LGVD = length gap Variolink dentin [%], LGV = sum length gap Variolink enamel and dentin [%].

Material	Interface	Parameter	t0	t1	t2	t3
**LS2**	1	LGVC	3.56	19.44	33.76	48.38
			[1.98; 6.28]	[17.36; 24.45]	[29.92; 36.58]	[42.50; 52.56]
	2	LGVE	15.52	10.70	19.64	26.15
			[12.34; 18.24]	[6.28; 14.15]	[14.98; 28.77]	[18.48; 43.78]
		LGVD	32.29	27.75	47.69	59.69
			[24.14; 44.02]	[19.84; 39.25]	[39.59; 59.74]	[50.12; 69.49]
		LGV	25.25	17.56	34.77	44.11
			[19.06; 34.86]	[12.69; 32.74]	[23.97; 49.64]	[30.96; 55.58]
**RBC**	1	LGVC	16.70	2.33	3.98	5.20
			[10.91; 18.70]	[0.55; 4.02]	[1.92; 5.68]	[3.99; 7.21]
	2	LGVE	10.03	18.45	32.98	36.87
			[7.81; 15.42]	[11.16; 21.58]	[18.95; 34.87]	[28.17; 45.41]
		LGVD	28.35	34.56	42.33	52.47
			[25.55; 29.84]	[28.00; 40.43]	[36.90; 54.25]	[45.17; 58.71]
		LGV	20.45	25.07	36.04	43.86
			[17.22; 23.83]	[21.95; 29.45]	[29.20; 38.33]	[37.07; 45.64]

## Data Availability

Data supporting reported results are given in the respective tables and Supplementary Information 1, 2 and 3.

## Supplementary Materials

The following supporting information can be downloaded at https://www.mdpi.com/article/10.3390/dj14020116/s1. File S1: Detailed description of the statistical analyses. File S2: Detailed description of the power analysis. Table S1: Materials, devices, and software used in the study.

## Abbreviations

The following abbreviations are used in this manuscript:

LS2	lithium disilicate ceramic
RBC	nanohybrid composite
TCML	thermomechanical loading
FEA	finite element analyses
SD-OCT	spectral-domain optical coherence tomography
LCI	length composite interface
LEI	length enamel interface
LDI	length dentin interface
LGVC	length gap Variolink composite/ceramic
LGVE	length gap Variolink enamel
LGVD	length gap Variolink dentin
LGV	length gap Variolink
