# Supplementary material for: Gap Formation at Luting Interfaces of CAD/CAM Ceramic and Composite Partial Crowns Assessed by OCT
Source: Dent J (Basel). 2026 Feb 17;14(2):116. doi: 10.3390/dj14020116 (PMC12939042; doi:10.3390/dj14020116)
Supplement: Supplementary file 1 [file dentistry-14-00116-s001.zip › dentistry-4054609-supplementary.pdf]

Supplement file S1: Detailed description of the statistical analysis (sample randomization, data preparation, summary statistics, test statistics), R code by Tobias Meißner and Marco Pellino.

S1. Sample Randomization Procedure

The 16 teeth were randomly distributed into two experimental groups (LS2 and RBC) using a systematic randomization approach:

```
# Load sample IDs from CSV file
sample_ids <- read.csv("Sample_ID.csv")
specimen_ids <- sample_ids$SPEC_ID

# Set seed for reproducibility
set.seed(42)

# Random permutation of all specimen IDs
randomized_ids <- sample(specimen_ids)

# Split into two groups of 8 specimens each
group_assignment <- data.frame(
  SPEC_ID = randomized_ids,
  Group = rep(c("LS2", "RBC"), each = 8)
)

# Verify equal distribution
table(group_assignment$Group)
```

This approach ensures: - Complete randomization of specimen allocation - Equal group sizes (8 specimens per group) - Reproducible randomization through seed setting - Traceability of original specimen IDs

S2. Data Preparation

S2.1 Data Loading

```
raw_data <- read.csv(here::here("IMT_Palsa_MainTable.CSV"), sep = ";", dec = ",")
```

S2.2 Data Transformation

We transform the data to only include the relevant columns and filter for OCT. The relevant measurements are:

- LGVC = length gap Variolink composite / ceramic,
- LGVE = length gap Variolink enamel,
- LGVD = length gap Variolink dentin,
- LGV = sum length gap Variolink enamel + dentin (LGVE + LGVD)

```
factors <- c("SPEC_ID", "Group", "Samplenumber", "Material", "Time")
measurements <- c("LGVC", "LGVE", "LGVD", "LGV")

data <- raw_data %>%
    dplyr::filter(Methode == "OCT" & Group %in% c("C", "D")) %>%
    dplyr::mutate(Group = case_when(Group == "C" ~ "LS2",
                                    Group == "D" ~ "RBC")) %>%
    dplyr::rename(LGV = LGVB) %>%
    dplyr::select(SPEC_ID,
                  Samplenumber,
                  Toothposition,
                  Group,
                  Material,
                  Time,
                  LGVC,
                  LGVE,
                  LGVD,
                  LGV) %>%
    dplyr::mutate(across(all_of(factors),factor)) %>%
    tidyr::unite( "UID", c("Group", "Samplenumber", "Toothposition"),
                  remove = FALSE) %>%
    tidyr::gather(key = "Measurement",
                  value= "Value",
                  all_of(measurements)) %>%
```

```
    dplyr::group_by(Group, Time, Measurement) %>%
    dplyr::mutate(
      is_outlier = case_when(Value >quantile(Value)[4] + 1.5*IQR(Value) ~ 4,
                             Value < quantile(Value)[2] - 1.5*IQR(Value) ~ 4,
                             TRUE ~ 21)
                  ) %>%
    droplevels() %>%
    dplyr::ungroup() %>%
    dplyr::mutate(across(all_of(c("UID", "Measurement")),factor)) %>%
    dplyr::mutate(Group_Time = interaction(Group, Time, drop = TRUE)) %>%
    droplevels()
```

S3. Summary Statistics

```
data %>%
  dplyr::filter(Measurement == "LGVC") %>%
  dplyr::group_by(Group, Time, Measurement) %>%
  rstatix::get_summary_stats(Value, type ="common") %>%
  dplyr::left_join(
    data %>%
      dplyr::filter(Measurement == "LGVC") %>%
      dplyr::group_by(Group, Time) %>%
      dplyr::summarise(q1 = quantile(Value, 0.25),
                       q3 = quantile(Value, 0.75),
                       .groups = "drop"),
    by = c("Group", "Time")
  ) %>%
  dplyr::select(-c(Measurement, variable)) %>%
  dplyr::mutate(across(where(is.numeric), round, 2))
```

Table S1: Summary Statistics: LGVC

Group	Time	n	min	max	median	iqr	mean	sd	se	ci	q1	q3
LS2	t0	8	0.74	9.93	3.56	4.30	4.34	3.21	1.13	2.68	1.98	6.28
LS2	t1	8	1.58	36.46	19.44	7.10	20.41	10.08	3.56	8.43	17.36	24.45
LS2	t2	8	6.79	46.68	33.76	6.66	32.15	11.78	4.16	9.85	29.92	36.58
LS2	t3	8	9.02	93.28	48.38	10.06	48.73	23.07	8.16	19.29	42.50	52.56
RBC	t0	8	7.65	31.51	16.70	7.79	16.64	8.03	2.84	6.72	10.91	18.70
RBC	t1	8	0.38	8.16	2.33	3.47	2.82	2.70	0.96	2.26	0.55	4.02
RBC	t2	8	1.10	10.55	3.98	3.76	4.36	3.14	1.11	2.63	1.92	5.68
RBC	t3	8	2.76	15.44	5.20	3.22	6.32	4.04	1.43	3.38	3.99	7.21

```
data %>%
  dplyr::filter(Measurement == "LGVE") %>%
  dplyr::group_by(Group, Time, Measurement) %>%
  rstatix::get_summary_stats(Value, type ="common") %>%
  dplyr::left_join(
    data %>%
      dplyr::filter(Measurement == "LGVE") %>%
      dplyr::group_by(Group, Time) %>%
      dplyr::summarise(q1 = quantile(Value, 0.25),
                       q3 = quantile(Value, 0.75), .groups = "drop"),
    by = c("Group", "Time")
  ) %>%
  dplyr::select(-c(Measurement, variable)) %>%
  dplyr::mutate(across(where(is.numeric), round, 2))
```

Table S2: Summary Statistics: LGVE

Group	Time	n	min	max	median	iqr	mean	sd	se	ci	q1	q3
LS2	t0	8	5.62	33.06	15.52	5.90	16.28	8.23	2.91	6.88	12.34	18.24
LS2	t1	8	4.96	23.28	10.70	7.87	11.71	6.63	2.34	5.54	6.28	14.15
LS2	t2	8	12.07	45.21	19.64	13.79	23.06	11.21	3.96	9.37	14.98	28.77
LS2	t3	8	17.48	52.78	26.15	25.30	31.06	14.17	5.01	11.85	18.48	43.78
RBC	t0	8	5.14	24.04	10.03	7.62	12.24	6.70	2.37	5.60	7.81	15.42
RBC	t1	8	4.00	26.22	18.45	10.42	16.54	7.55	2.67	6.31	11.16	21.58

RBC	t2	8	11.02	39.40	32.98	15.92	27.97	10.27	3.63	8.59	18.95	34.87
RBC	t3	8	14.12	46.62	36.87	17.25	35.11	11.70	4.14	9.78	28.17	45.41

```
data %>%
  dplyr::filter(Measurement == "LGVD") %>%
  dplyr::group_by(Group, Time, Measurement) %>%
  rstatix::get_summary_stats(Value, type ="common") %>%
  dplyr::left_join(
    data %>%
      dplyr::filter(Measurement == "LGVD") %>%
      dplyr::group_by(Group, Time) %>%
      dplyr::summarise(q1 = quantile(Value, 0.25),
                       q3 = quantile(Value, 0.75), .groups = "drop"),
    by = c("Group", "Time")
  ) %>%
  dplyr::select(-c(Measurement, variable)) %>%
  dplyr::mutate(across(where(is.numeric), round, 2))
```

Table S3: Summary Statistics: LGVD

Group	Time	n	min	max	median	iqr	mean	sd	se	ci	q1	q3
LS2	t0	8	17.20	61.67	32.29	19.88	35.11	15.78	5.58	13.19	24.14	44.02
LS2	t1	8	14.03	64.09	27.75	19.41	32.81	18.91	6.69	15.81	19.84	39.25
LS2	t2	8	24.94	71.79	47.69	20.15	48.64	16.93	5.98	14.15	39.59	59.74
LS2	t3	8	23.73	83.32	59.69	19.38	57.36	20.93	7.40	17.50	50.12	69.49
RBC	t0	8	9.62	42.67	28.35	4.29	27.38	9.08	3.21	7.59	25.55	29.84
RBC	t1	8	14.36	56.88	34.56	12.43	34.76	12.85	4.54	10.74	28.00	40.43
RBC	t2	8	15.81	63.88	42.33	17.35	42.80	15.53	5.49	12.98	36.90	54.25
RBC	t3	8	31.36	73.94	52.47	13.54	51.70	13.47	4.76	11.26	45.17	58.71

```
data %>%
  dplyr::filter(Measurement == "LGV") %>%
  dplyr::group_by(Group, Time, Measurement) %>%
  rstatix::get_summary_stats(Value, type ="common") %>%
  dplyr::left_join(
    data %>%
      dplyr::filter(Measurement == "LGV") %>%
      dplyr::group_by(Group, Time) %>%
      dplyr::summarise(q1 = quantile(Value, 0.25),
                       q3 = quantile(Value, 0.75), .groups = "drop"),
    by = c("Group", "Time")
  ) %>%
  dplyr::select(-c(Measurement, variable)) %>%
  dplyr::mutate(across(where(is.numeric), round, 2))
```

Table S4: Summary Statistics: LGV

Group	Time	n	min	max	median	iqr	mean	sd	se	ci	q1	q3
LS2	t0	8	10.90	49.28	25.25	15.80	27.42	13.35	4.72	11.16	19.06	34.86
LS2	t1	8	11.75	50.69	17.56	20.06	24.58	15.58	5.51	13.03	12.69	32.74
LS2	t2	8	19.57	57.57	34.77	25.67	36.97	15.26	5.39	12.76	23.97	49.64
LS2	t3	8	21.44	69.44	44.10	24.62	44.45	18.54	6.55	15.50	30.96	55.58
RBC	t0	8	11.18	28.22	20.44	6.61	20.02	5.60	1.98	4.68	17.22	23.83
RBC	t1	8	12.16	39.91	25.07	7.51	25.21	8.57	3.03	7.16	21.95	29.45
RBC	t2	8	17.88	52.10	36.04	9.13	34.37	10.31	3.64	8.62	29.20	38.33
RBC	t3	8	25.63	60.98	43.85	8.57	41.92	11.55	4.08	9.66	37.07	45.64

S4. Statistical Analysis

S4.1 Normality Testing

Shapiro-Wilk test for normality assessment ($p > 0.05$ indicates normal distribution).

```
data %>%
  dplyr::group_by(Group, Time, Measurement) %>%
  rstatix::shapiro_test(Value) %>%
  dplyr::mutate(p = round(p, 4)) %>%
  dplyr::arrange(Measurement, Group, Time)
```

Table S5: Shapiro-Wilk Normality Test

Group	Time	Measurement	variable	statistic	p
LS2	t0	LGV	Value	0.9539677	0.7511
LS2	t1	LGV	Value	0.8088765	0.0356
LS2	t2	LGV	Value	0.9037915	0.3124
LS2	t3	LGV	Value	0.9200547	0.4303
RBC	t0	LGV	Value	0.9677322	0.8796
RBC	t1	LGV	Value	0.9672949	0.8760
RBC	t2	LGV	Value	0.9652782	0.8586
RBC	t3	LGV	Value	0.9177765	0.4121
LS2	t0	LGVC	Value	0.9371285	0.5831
LS2	t1	LGVC	Value	0.9354093	0.5666
LS2	t2	LGVC	Value	0.8605890	0.1217
LS2	t3	LGVC	Value	0.8861691	0.2155
RBC	t0	LGVC	Value	0.9192211	0.4236
RBC	t1	LGVC	Value	0.8657223	0.1368
RBC	t2	LGVC	Value	0.8921088	0.2448
RBC	t3	LGVC	Value	0.7920729	0.0235
LS2	t0	LGVD	Value	0.9348475	0.5612
LS2	t1	LGVD	Value	0.8542711	0.1052
LS2	t2	LGVD	Value	0.9497239	0.7084
LS2	t3	LGVD	Value	0.9231077	0.4556
RBC	t0	LGVD	Value	0.8854722	0.2123
RBC	t1	LGVD	Value	0.9930764	0.9985
RBC	t2	LGVD	Value	0.9694769	0.8939
RBC	t3	LGVD	Value	0.9750908	0.9347
LS2	t0	LGVE	Value	0.9256492	0.4774
LS2	t1	LGVE	Value	0.8944819	0.2574
LS2	t2	LGVE	Value	0.8807959	0.1917
LS2	t3	LGVE	Value	0.8577200	0.1139
RBC	t0	LGVE	Value	0.9013639	0.2972
RBC	t1	LGVE	Value	0.9570331	0.7814
RBC	t2	LGVE	Value	0.8685543	0.1459
RBC	t3	LGVE	Value	0.9031498	0.3083

S4.2 Homogeneity of Variances

Levene's test for variance homogeneity ($p > 0.05$ indicates equal variances).

```
data %>%
  dplyr::group_by(Measurement) %>%
  rstatix::levene_test(Value ~ interaction(Group, Time)) %>%
  dplyr::mutate(p = round(p, 4))
```

Table S6: Levene's Test for Homogeneity of Variances

Measurement	df1	df2	statistic	p
LGV	7	56	1.7203979	0.1229
LGVC	7	56	1.9546472	0.0779
LGVD	7	56	0.7952916	0.5944
LGVE	7	56	1.1485716	0.3469

Note: Non-parametric tests used due to small sample size and some normality violations.

S4.3 Between-Group Comparisons

Mann-Whitney U test for independent samples (LS2 vs RBC at each timepoint).

```
# P-values
mann_whitney_p <- data %>%
  dplyr::group_by(Time, Measurement) %>%
```

```
  rstatix::wilcox_test(Value ~ Group, paired = FALSE) %>%
  dplyr::ungroup() %>%
  dplyr::group_by(Measurement) %>%
  dplyr::mutate(p.adj = p.adjust(p, method = "bonferroni")) %>%
  dplyr::ungroup()

# Effect sizes
mann_whitney_eff <- data %>%
  dplyr::group_by(Time, Measurement) %>%
  rstatix::wilcox_effsize(Value ~ Group, paired = FALSE) %>%
  dplyr::ungroup()

# Combined results
mann_whitney_combined <- mann_whitney_p %>%
  dplyr::left_join(mann_whitney_eff,
                   by = c("Time", "Measurement", "group1", "group2", "n1", "n2")) %>%
  dplyr::select(Measurement, Time, group1, group2, n1, n2, statistic, p, p.adj, effsize) %>
%
  dplyr::mutate(across(where(is.numeric), round, 4)) %>%
  dplyr::arrange(Measurement, Time)

mann_whitney_combined
```

Table S7: Mann-Whitney U Test: Between-Group Comparisons

Measurement	Time	group1	group2	n1	n2	statistic	p	p.adj	effsize
LGV	t0	LS2	RBC	8	8	44	0.2340	0.9360	0.3151
LGV	t1	LS2	RBC	8	8	26	0.5740	1.0000	0.1575
LGV	t2	LS2	RBC	8	8	36	0.7210	1.0000	0.1050
LGV	t3	LS2	RBC	8	8	34	0.8780	1.0000	0.0525
LGVC	t0	LS2	RBC	8	8	2	0.0006	0.0025	0.7877
LGVC	t1	LS2	RBC	8	8	60	0.0019	0.0074	0.7351
LGVC	t2	LS2	RBC	8	8	63	0.0003	0.0012	0.8139
LGVC	t3	LS2	RBC	8	8	63	0.0003	0.0012	0.8139
LGVD	t0	LS2	RBC	8	8	42	0.3280	1.0000	0.2626
LGVD	t1	LS2	RBC	8	8	27	0.6450	1.0000	0.1313
LGVD	t2	LS2	RBC	8	8	39	0.5050	1.0000	0.1838
LGVD	t3	LS2	RBC	8	8	40	0.4420	1.0000	0.2100
LGVE	t0	LS2	RBC	8	8	43	0.2790	1.0000	0.2888
LGVE	t1	LS2	RBC	8	8	23	0.3820	1.0000	0.2363
LGVE	t2	LS2	RBC	8	8	21	0.2790	1.0000	0.2888
LGVE	t3	LS2	RBC	8	8	25	0.5050	1.0000	0.1838

S4.4 Within-Group Comparisons

Friedman test for timepoint effects within each material (repeated measures).

```
data %>%
  dplyr::group_by(Group, Measurement) %>%
  rstatix::friedman_test(Value ~ Time | UID) %>%
  dplyr::mutate(p = round(p, 4)) %>%
  dplyr::select(Measurement, Group, df, statistic, p)
```

Table S8: Friedman Test: Timepoint Effects Within Groups

Measurement	Group	df	statistic	p
LGV	LS2	3	20.25	2e-04
LGVC	LS2	3	22.95	0e+00
LGVD	LS2	3	16.20	1e-03
LGVE	LS2	3	22.95	0e+00
LGV	RBC	3	24.00	0e+00
LGVC	RBC	3	24.00	0e+00
LGVD	RBC	3	20.85	1e-04
LGVE	RBC	3	21.15	1e-04

Conover post-hoc test for Friedman (repeated measures - timepoints).

```r
# Effect sizes for paired comparisons
wilcox_paired_eff <- data %>%
  dplyr::group_by(Group, Measurement) %>%
  rstatix::wilcox_effsize(Value ~ Time, paired = TRUE) %>%
  dplyr::ungroup()

conover_results <- data %>%
  dplyr::group_by(Group, Measurement) %>%
  dplyr::do({
    test_data <- .
    "conover_test" <- DescTools::ConoverTest(Value ~ Time, data = test_data, method = "bonf
erroni")
    result_df <- as.data.frame(conover_test[[1]])

    result_df %>%
      dplyr::mutate(
        Measurement = unique(test_data$Measurement),
        Group = unique(test_data$Group),
        comparison = gsub("[^t0-9-]", "", rownames(.)),
        group1 = stringr::str_extract(comparison, "t[0-9]+"),
        group2 = stringr::str_extract(comparison, "-t[0-9]+$") %>% stringr::str_remove("-")
,
        group1_num = as.numeric(stringr::str_remove(group1, "t")),
        group2_num = as.numeric(stringr::str_remove(group2, "t")),
        temp_group1 = ifelse(group1_num > group2_num, group2, group1),
        temp_group2 = ifelse(group1_num > group2_num, group1, group2),
        group1 = temp_group1,
        group2 = temp_group2,
        pval = as.numeric(format(pval, scientific = FALSE, digits = 4))
      )
  }) %>%
  dplyr::rename(p.adj = pval) %>%
  dplyr::ungroup() %>%
  dplyr::left_join(wilcox_paired_eff,
                   by = c("Group", "Measurement", "group1", "group2")) %>%
  dplyr::mutate(across(where(is.numeric), round, 4)) %>%
  dplyr::select(Measurement, Group, group1, group2, n1, n2, `mean rank diff`, p.adj,
                effsize, -group1_num, -group2_num, -temp_group1, -temp_group2, -comparison,
 -.y.)

conover_results
```

Table S9: Conover Post-Hoc Test: Pairwise Comparisons for Timepoint Effects Within Groups

Measurement	Group	group1	group2	n1	n2	mean rank diff	p.adj	effsize
LGV	LS2	t0	t1	8	8	-1.875	1.0000	0.4456
LGV	LS2	t0	t2	8	8	5.625	1.0000	0.8416
LGV	LS2	t0	t3	8	8	9.250	0.2493	0.8911
LGV	LS2	t1	t2	8	8	7.500	0.5657	0.8911
LGV	LS2	t1	t3	8	8	11.125	0.0949	0.8911
LGV	LS2	t2	t3	8	8	3.625	1.0000	0.8911
LGVC	LS2	t0	t1	8	8	8.500	0.0309	0.8416
LGVC	LS2	t0	t2	8	8	14.375	0.0001	0.8911
LGVC	LS2	t0	t3	8	8	20.625	0.0000	0.8911
LGVC	LS2	t1	t2	8	8	5.875	0.2703	0.8911
LGVC	LS2	t1	t3	8	8	12.125	0.0010	0.8911
LGVC	LS2	t2	t3	8	8	6.250	0.2028	0.8911
LGVD	LS2	t0	t1	8	8	-1.000	1.0000	0.2475
LGVD	LS2	t0	t2	8	8	6.500	0.8726	0.8416
LGVD	LS2	t0	t3	8	8	9.500	0.2227	0.8416

LGVD	LS2	t1	t2	8	8	7.500	0.5701	0.8911
LGVD	LS2	t1	t3	8	8	10.500	0.1338	0.8911
LGVD	LS2	t2	t3	8	8	3.000	1.0000	0.8416
LGVE	LS2	t0	t1	8	8	-4.625	1.0000	0.8416
LGVE	LS2	t0	t2	8	8	5.000	1.0000	0.8911
LGVE	LS2	t0	t3	8	8	10.625	0.0649	0.8911
LGVE	LS2	t1	t2	8	8	9.625	0.1182	0.8911
LGVE	LS2	t1	t3	8	8	15.250	0.0031	0.8911
LGVE	LS2	t2	t3	8	8	5.625	0.9563	0.8911
LGV	RBC	t0	t1	8	8	5.375	0.7726	0.8911
LGV	RBC	t0	t2	8	8	12.125	0.0087	0.8911
LGV	RBC	t0	t3	8	8	17.500	0.0001	0.8911
LGV	RBC	t1	t2	8	8	6.750	0.3560	0.8911
LGV	RBC	t1	t3	8	8	12.125	0.0087	0.8911
LGV	RBC	t2	t3	8	8	5.375	0.7726	0.8911
LGVC	RBC	t0	t1	8	8	-18.750	0.0000	0.8911
LGVC	RBC	t0	t2	8	8	-14.750	0.0005	0.8911
LGVC	RBC	t0	t3	8	8	-11.000	0.0120	0.8911
LGVC	RBC	t1	t2	8	8	4.000	1.0000	0.8911
LGVC	RBC	t1	t3	8	8	7.750	0.1396	0.8911
LGVC	RBC	t2	t3	8	8	3.750	1.0000	0.8911
LGVD	RBC	t0	t1	8	8	5.500	1.0000	0.8416
LGVD	RBC	t0	t2	8	8	10.375	0.0785	0.8416
LGVD	RBC	t0	t3	8	8	15.125	0.0036	0.8911
LGVD	RBC	t1	t2	8	8	4.875	1.0000	0.8911
LGVD	RBC	t1	t3	8	8	9.625	0.1224	0.8911
LGVD	RBC	t2	t3	8	8	4.750	1.0000	0.8911
LGVE	RBC	t0	t1	8	8	3.875	1.0000	0.5446
LGVE	RBC	t0	t2	8	8	12.125	0.0102	0.8911
LGVE	RBC	t0	t3	8	8	16.500	0.0004	0.8911
LGVE	RBC	t1	t2	8	8	8.250	0.1521	0.8911
LGVE	RBC	t1	t3	8	8	12.625	0.0070	0.8911
LGVE	RBC	t2	t3	8	8	4.375	1.0000	0.8416

S5. Session Information

```
sessionInfo()

R version 4.4.1 (2024-06-14 ucrt)

other attached packages:
[1] purrr_1.0.2       DescTools_0.99.54 rstatix_0.7.2     tidyr_1.3.1
[5] dplyr_1.1.4       here_1.0.1
```

S1. Overview

This power analysis evaluates sample size requirements for comparing gap percentages between two material groups (A and B) across 4 timepoints using simulation methods appropriate for non-normally distributed data.

Study Design:

- **Groups:** 2 (Group A vs Group B)
- **Outcome:** Gap percentage measurements at material interfaces (0-100%)
- **Timepoints:** 4 repeated measurements per sample
- **Expected difference:** 1-5% difference between groups
- **Statistical test:** Kruskal-Wallis test (non-parametric)
- **Target power:** 80% at α = 0.05

S2. Setup

```
rm(list=ls())
Sys.setenv(LANG = "en")
options(scipen = 999)

install.load.package <- function(x) {
  if (!require(x, character.only = TRUE))
    install.packages(x)
  require(x, character.only = TRUE)
}

package_vec <- c(
  "tidyverse",
  "knitr",
  "kableExtra",
  "MASS",
  "parallel",
  "doParallel",
  "foreach",
  "conflicted"
)

sapply(package_vec, install.load.package)

 tidyverse      knitr kableExtra       MASS   parallel doParallel    foreach
      TRUE       TRUE       TRUE       TRUE       TRUE       TRUE       TRUE
conflicted
      TRUE

library(conflicted)
conflict_prefer("select", "dplyr")
conflict_prefer("filter", "dplyr")

save_plots <- TRUE

set.seed(42)

n_cores <- parallel::detectCores() - 1
n_cores <- max(1, n_cores)

R.version.string

[1] "R version 4.4.1 (2024-06-14 ucrt)"
```

S2.1 Simulation Parameters

We run 1000 simulations per condition to estimate power and use sample sizes from 5 to 15 and effect sizes from 1% to 5%. Four timepoints are simulated for each sample, as well as a within-subject correlation of 0.6. A group A mean of 10% and a standard deviation of 5% are used.

```r
n_sims <- 1000
alpha <- 0.05
target_power <- 0.8

sample_sizes <- seq(5, 15, by = 1)
effect_sizes <- c(1,2,3,4,5) / 100

n_timepoints <- 4

group_a_mean <- 0.10
group_a_sd <- 0.05

within_subject_cor <- 0.6
```

S2.2 Simulation Functions

This section defines the functions used for simulating data and estimating power.

```r
estimate_beta_params <- function(mean_val, sd_val) {
  mean_val <- pmax(0.001, pmin(0.999, mean_val))
  variance <- sd_val^2
  max_var <- mean_val * (1 - mean_val)
  variance <- pmin(variance, max_var * 0.99)

  alpha_param <- mean_val * ((mean_val * (1 - mean_val) / variance) - 1)
  beta_param <- (1 - mean_val) * ((mean_val * (1 - mean_val) / variance) - 1)

  alpha_param <- pmax(0.5, alpha_param)
  beta_param <- pmax(0.5, beta_param)

  return(list(alpha = alpha_param, beta = beta_param))
}

generate_correlated_beta <- function(n_subjects, n_timepoints, mean_val, sd_val,
                                     within_cor, seed_offset = 0) {
  set.seed(42 + seed_offset)

  sigma_matrix <- matrix(within_cor, nrow = n_timepoints, ncol = n_timepoints)
  diag(sigma_matrix) <- 1

  normal_data <- mvrnorm(n = n_subjects, mu = rep(0, n_timepoints), Sigma = sigma_matrix)

  uniform_data <- pnorm(normal_data)

  beta_params <- estimate_beta_params(mean_val, sd_val)
  beta_data <- qbeta(uniform_data, shape1 = beta_params$alpha, shape2 = beta_params$beta)

  beta_data <- pmax(0, pmin(1, beta_data))

  return(beta_data * 100)
}

simulate_study <- function(n_per_group, effect_size, n_timepoints,
                           group_a_mean, group_a_sd, within_cor, sim_id) {
  group_b_mean <- group_a_mean + effect_size
  group_b_mean <- pmin(group_b_mean, 0.95)

  data_a <- generate_correlated_beta(
    n_subjects = n_per_group,
    n_timepoints = n_timepoints,
    mean_val = group_a_mean,
    sd_val = group_a_sd,
    within_cor = within_cor,
    seed_offset = sim_id * 2
  )

  data_b <- generate_correlated_beta(
    n_subjects = n_per_group,
    n_timepoints = n_timepoints,
```

```r
    mean_val = group_b_mean,
    sd_val = group_a_sd,
    within_cor = within_cor,
    seed_offset = sim_id * 2 + 1
  )

  df_a <- data.frame(
    subject_id = rep(1:n_per_group, each = n_timepoints),
    group = "A",
    timepoint = rep(1:n_timepoints, n_per_group),
    gap_pct = as.vector(t(data_a))
  )

  df_b <- data.frame(
    subject_id = rep((n_per_group + 1):(2 * n_per_group), each = n_timepoints),
    group = "B",
    timepoint = rep(1:n_timepoints, n_per_group),
    gap_pct = as.vector(t(data_b))
  )

  df <- rbind(df_a, df_b)
  df$group <- factor(df$group)
  df$timepoint <- factor(df$timepoint)

  return(df)
}

run_kruskal_test <- function(df) {
  p_values <- numeric(length(unique(df$timepoint)))

  for (tp in unique(df$timepoint)) {
    df_tp <- df[df$timepoint == tp, ]
    test_result <- kruskal.test(gap_pct ~ group, data = df_tp)
    p_values[as.numeric(tp)] <- test_result$p.value
  }

  return(p_values)
}

run_single_sim <- function(sim_id, n_per_group, effect_size, n_timepoints,
                           group_a_mean, group_a_sd, within_cor, alpha) {
  df <- simulate_study(
    n_per_group = n_per_group,
    effect_size = effect_size,
    n_timepoints = n_timepoints,
    group_a_mean = group_a_mean,
    group_a_sd = group_a_sd,
    within_cor = within_cor,
    sim_id = sim_id
  )

  p_values <- run_kruskal_test(df)

  list(
    sig_any = as.numeric(any(p_values < alpha)),
    sig_all = as.numeric(all(p_values < alpha)),
    sig_per_tp = as.numeric(p_values < alpha)
  )
}

run_power_simulation_parallel <- function(n_per_group, effect_size, n_sims, n_timepoints,
                                          group_a_mean, group_a_sd, within_cor, alpha,
                                          n_cores) {

  cl <- makeCluster(n_cores)
  registerDoParallel(cl)
```

```
  clusterExport(cl, c("simulate_study", "run_kruskal_test", "generate_correlated_beta",
                      "estimate_beta_params", "run_single_sim", "mvrnorm"),
              envir = environment())

  results <- foreach(sim = 1:n_sims, .combine = rbind, .packages = c("MASS")) %dopar% {
    res <- run_single_sim(
      sim_id = sim,
      n_per_group = n_per_group,
      effect_size = effect_size,
      n_timepoints = n_timepoints,
      group_a_mean = group_a_mean,
      group_a_sd = group_a_sd,
      within_cor = within_cor,
      alpha = alpha
    )
    c(res$sig_any, res$sig_all, res$sig_per_tp)
  }

  stopCluster(cl)

  significant_any <- results[, 1]
  significant_all <- results[, 2]
  significant_per_tp <- results[, 3:(2 + n_timepoints)]

  power_any <- mean(significant_any)
  power_all <- mean(significant_all)
  power_per_tp <- colMeans(significant_per_tp)

  ci_any <- binom.test(sum(significant_any), n_sims, conf.level = 0.95)$conf.int
  ci_all <- binom.test(sum(significant_all), n_sims, conf.level = 0.95)$conf.int

  ci_per_tp <- lapply(1:n_timepoints, function(tp) {
    binom.test(sum(significant_per_tp[, tp]), n_sims, conf.level = 0.95)$conf.int
  })

  return(list(
    n_per_group = n_per_group,
    effect_size = effect_size,
    power_any = power_any,
    power_any_ci_lower = ci_any[1],
    power_any_ci_upper = ci_any[2],
    power_all = power_all,
    power_all_ci_lower = ci_all[1],
    power_all_ci_upper = ci_all[2],
    power_per_tp = power_per_tp,
    ci_per_tp = ci_per_tp
  ))
}
```

S3. Run Power Analysis

This section runs the power analysis simulations. We use the parameters defined above to run the simulations. Since the computation is heavy, we use parallel processing.

```
param_grid <- expand.grid(
  n_per_group = sample_sizes,
  effect_size = effect_sizes
)

results_list <- lapply(1:nrow(param_grid), function(i) {
  run_power_simulation_parallel(
    n_per_group = param_grid$n_per_group[i],
    effect_size = param_grid$effect_size[i],
    n_sims = n_sims,
    n_timepoints = n_timepoints,
    group_a_mean = group_a_mean,
    group_a_sd = group_a_sd,
    within_cor = within_subject_cor,
```

```
    alpha = alpha,
    n_cores = n_cores
  )
})
```

S4. Results

S4.1 Main Power Results Table

The following table shows the power estimates for detecting group differences at any timepoint with the corresponding sample sizes.

```
results_df <- do.call(rbind, lapply(results_list, function(x) {
  data.frame(
    n_per_group = x$n_per_group,
    effect_size_pct = x$effect_size * 100,
    power = x$power_any,
    ci_lower = x$power_any_ci_lower,
    ci_upper = x$power_any_ci_upper
  )
}))

results_wide <- results_df %>%
  mutate(
    power_ci = sprintf("%.1f%% [%.1f%%, %.1f%%]",
                       power * 100, ci_lower * 100, ci_upper * 100),
    effect_label = factor(paste0(effect_size_pct, "% Effect"),
                          levels = paste0(sort(unique(effect_size_pct)), "% Effect"))
  ) %>%
  select(n_per_group, effect_label, power_ci) %>%
  pivot_wider(names_from = effect_label, values_from = power_ci)

n_effects <- ncol(results_wide) - 1

kable(results_wide,
      align = rep("c", ncol(results_wide)),
      caption = "Power estimates for detecting group differences at any timepoint")
```

Table S10: Power estimates for detecting group differences at any timepoint

Power estimates for detecting group differences at any timepoint

n_per_group	1% Effect	2% Effect	3% Effect	4% Effect	5% Effect
5	19.1% [16.7%, 21.7%]	24.9% [22.2%, 27.7%]	35.8% [32.8%, 38.9%]	48.0% [44.9%, 51.1%]	61.2% [58.1%, 64.2%]
6	36.4% [33.4%, 39.5%]	44.2% [41.1%, 47.3%]	55.4% [52.3%, 58.5%]	65.9% [62.9%, 68.8%]	77.2% [74.5%, 79.8%]
7	18.8% [16.4%, 21.4%]	28.1% [25.3%, 31.0%]	43.1% [40.0%, 46.2%]	58.9% [55.8%, 62.0%]	73.9% [71.1%, 76.6%]
8	73.9% [71.1%, 76.6%]	80.5% [77.9%, 82.9%]	87.7% [85.5%, 89.7%]	93.5% [91.8%, 94.9%]	96.6% [95.3%, 97.6%]
9	22.4% [19.9%, 25.1%]	32.4% [29.5%, 35.4%]	49.5% [46.4%, 52.6%]	66.2% [63.2%, 69.1%]	82.0% [79.5%, 84.3%]
10	41.9% [38.8%, 45.0%]	54.9% [51.8%, 58.0%]	70.3% [67.4%, 73.1%]	84.1% [81.7%, 86.3%]	92.6% [90.8%, 94.1%]
11	20.5% [18.0%, 23.1%]	35.6% [32.6%, 38.7%]	56.7% [53.6%, 59.8%]	73.8% [71.0%, 76.5%]	87.9% [85.7%, 89.9%]
12	73.6% [70.8%, 76.3%]	82.7% [80.2%, 85.0%]	89.9% [87.9%, 91.7%]	95.7% [94.3%, 96.9%]	98.6% [97.7%, 99.2%]
13	21.2% [18.7%, 23.9%]	40.4% [37.3%, 43.5%]	63.6% [60.5%, 66.6%]	83.0% [80.5%, 85.3%]	92.7% [90.9%, 94.2%]
14	42.8% [39.7%, 45.9%]	61.0% [57.9%, 64.0%]	78.2% [75.5%, 80.7%]	90.5% [88.5%, 92.2%]	96.5% [95.2%, 97.6%]
15	22.2% [19.7%, 24.9%]	44.6% [41.5%, 47.7%]	68.5% [65.5%, 71.4%]	85.1% [82.7%, 87.3%]	95.4% [93.9%, 96.6%]

S4.2 Power at Each Timepoint

The following table shows the power estimates for detecting group differences at each timepoint with the corresponding sample sizes.

```r
max_effect <- max(effect_sizes)
max_effect_pct <- max_effect * 100

tp_results <- do.call(rbind, lapply(results_list, function(x) {
  if (abs(x$effect_size - max_effect) < 0.001) {
    data.frame(
      n_per_group = x$n_per_group,
      tp1_power = x$power_per_tp[1],
      tp1_ci_l = x$ci_per_tp[[1]][1],
      tp1_ci_u = x$ci_per_tp[[1]][2],
      tp2_power = x$power_per_tp[2],
      tp2_ci_l = x$ci_per_tp[[2]][1],
      tp2_ci_u = x$ci_per_tp[[2]][2],
      tp3_power = x$power_per_tp[3],
      tp3_ci_l = x$ci_per_tp[[3]][1],
      tp3_ci_u = x$ci_per_tp[[3]][2],
      tp4_power = x$power_per_tp[4],
      tp4_ci_l = x$ci_per_tp[[4]][1],
      tp4_ci_u = x$ci_per_tp[[4]][2]
    )
  }
}))

tp_display <- tp_results %>%
  mutate(
    `Timepoint 1` = sprintf("%.1f%% [%.1f%%, %.1f%%]",
                            tp1_power * 100, tp1_ci_l * 100, tp1_ci_u * 100),
    `Timepoint 2` = sprintf("%.1f%% [%.1f%%, %.1f%%]",
                            tp2_power * 100, tp2_ci_l * 100, tp2_ci_u * 100),
    `Timepoint 3` = sprintf("%.1f%% [%.1f%%, %.1f%%]",
                            tp3_power * 100, tp3_ci_l * 100, tp3_ci_u * 100),
    `Timepoint 4` = sprintf("%.1f%% [%.1f%%, %.1f%%]",
                            tp4_power * 100, tp4_ci_l * 100, tp4_ci_u * 100)
  ) %>%
  select(n_per_group, `Timepoint 1`, `Timepoint 2`, `Timepoint 3`, `Timepoint 4`)

kable(tp_display,
      col.names = c("n per Group", "Timepoint 1", "Timepoint 2",
                    "Timepoint 3", "Timepoint 4"),
      align = "c",
      caption = sprintf("Power estimates by timepoint for %.1f%% effect size", max_effect_p
ct))
```

Table S11: Power estimates by timepoint for 5.0% effect size

n per Group	Timepoint 1	Timepoint 2	Timepoint 3	Timepoint 4
5	30.2% [27.4%, 33.2%]	31.6% [28.7%, 34.6%]	31.7% [28.8%, 34.7%]	32.0% [29.1%, 35.0%]
6	37.5% [34.5%, 40.6%]	41.0% [37.9%, 44.1%]	37.8% [34.8%, 40.9%]	39.7% [36.7%, 42.8%]
7	43.5% [40.4%, 46.6%]	44.4% [41.3%, 47.5%]	43.1% [40.0%, 46.2%]	41.5% [38.4%, 44.6%]
8	55.1% [52.0%, 58.2%]	56.3% [53.2%, 59.4%]	55.0% [51.9%, 58.1%]	57.4% [54.3%, 60.5%]
9	53.3% [50.2%, 56.4%]	54.4% [51.3%, 57.5%]	52.1% [49.0%, 55.2%]	50.8% [47.7%, 53.9%]
10	59.2% [56.1%, 62.3%]	60.5% [57.4%, 63.5%]	58.1% [55.0%, 61.2%]	58.1% [55.0%, 61.2%]
11	60.6% [57.5%, 63.6%]	62.6% [59.5%, 65.6%]	62.7% [59.6%, 65.7%]	59.6% [56.5%, 62.7%]
12	64.5% [61.4%, 67.5%]	67.3% [64.3%, 70.2%]	65.3% [62.3%, 68.3%]	64.4% [61.3%, 67.4%]
13	72.2% [69.3%, 75.0%]	71.6% [68.7%, 74.4%]	70.7% [67.8%, 73.5%]	73.0% [70.1%, 75.7%]
14	72.1% [69.2%, 74.9%]	70.6% [67.7%, 73.4%]	71.4% [68.5%, 74.2%]	70.2% [67.3%, 73.0%]
15	77.7% [75.0%, 80.2%]	79.6% [77.0%, 82.1%]	77.5% [74.8%, 80.1%]	77.0% [74.3%, 79.6%]

S4.3 Sample Size Recommendations

We give a short overview of the minimum sample size per group to achieve 80% power for each effect size.

```
unique_effects <- sort(unique(results_df$effect_size_pct))

recommendations <- lapply(unique_effects, function(eff) {
  eff_data <- results_df[abs(results_df$effect_size_pct - eff) < 0.01, ]
  achieved <- eff_data[eff_data$power >= target_power, ]

  if (nrow(achieved) > 0) {
    best <- achieved[which.min(achieved$n_per_group), ]
    rec_text <- sprintf("n = %d (Power: %.1f%% [%.1f%%, %.1f%%])",
                        best$n_per_group, best$power * 100,
                        best$ci_lower * 100, best$ci_upper * 100)
  } else {
    max_row <- eff_data[which.max(eff_data$power), ]
    rec_text <- sprintf("n > 50 needed (Max power at n=%d: %.1f%%)",
                        max_row$n_per_group, max_row$power * 100)
  }

  data.frame(effect_size_pct = eff, recommendation = rec_text)
})

recommendations <- do.call(rbind, recommendations)

kable(recommendations,
      col.names = c("Effect Size (%)", "Minimum Sample Size Recommendation"),
      align = c("c", "l"),
      caption = "Minimum sample size per group to achieve 80% power")
```

Table S12: Minimum sample size per group to achieve 80% power

Minimum sample size per group to achieve 80% power

Effect Size (%)	Minimum Sample Size Recommendation
1	n > 50 needed (Max power at n=8: 73.9%)
2	n = 8 (Power: 80.5% [77.9%, 82.9%])
3	n = 8 (Power: 87.7% [85.5%, 89.7%])
4	n = 8 (Power: 93.5% [91.8%, 94.9%])
5	n = 8 (Power: 96.6% [95.3%, 97.6%])

S4.4 Power Curves

The following figure shows the power curves for different effect sizes after 1000 simulations.

```
effect_levels <- sort(unique(results_df$effect_size_pct))
n_effects_plot <- length(effect_levels)
color_palette <- scales::hue_pal()(n_effects_plot)
names(color_palette) <- as.character(effect_levels)
effect_labels <- paste0(effect_levels, "%")
names(effect_labels) <- as.character(effect_levels)

power_plot <- ggplot(results_df, aes(x = n_per_group, y = power,
                                     color = factor(effect_size_pct),
                                     fill = factor(effect_size_pct))) +
  geom_ribbon(aes(ymin = ci_lower, ymax = ci_upper), alpha = 0.2, color = NA) +
  geom_line(linewidth = 1.2) +
  geom_point(size = 2.5) +
  geom_hline(yintercept = target_power, linetype = "dashed", color = "red",
             linewidth = 0.8) +
  annotate("text", x = max(sample_sizes) - 5, y = target_power + 0.03,
           label = "80% Power Target", color = "red", size = 3.5) +
  scale_y_continuous(labels = scales::percent_format(),
                     limits = c(0, 1), breaks = seq(0, 1, 0.1)) +
  scale_x_continuous(breaks = sample_sizes) +
  scale_color_manual(values = color_palette, labels = effect_labels) +
  scale_fill_manual(values = color_palette, labels = effect_labels) +
```

```
  labs(
    x = "Sample Size per Group",
    y = "Statistical Power",
    color = "Effect Size",
    fill = "Effect Size",
    title = "Power Analysis: Gap Percentage Comparison",
    subtitle = "Kruskal-Wallis test with 4 repeated timepoints, α = 0.05"
  ) +
  theme_minimal(base_size = 12) +
  theme(
    legend.position = "bottom",
    panel.grid.minor = element_blank(),
    plot.title = element_text(face = "bold", size = 14),
    plot.subtitle = element_text(color = "gray40")
  )

print(power_plot)

if (save_plots) {
  ggsave("power_curves.png", power_plot, width = 10, height = 6, dpi = 600)
}
```

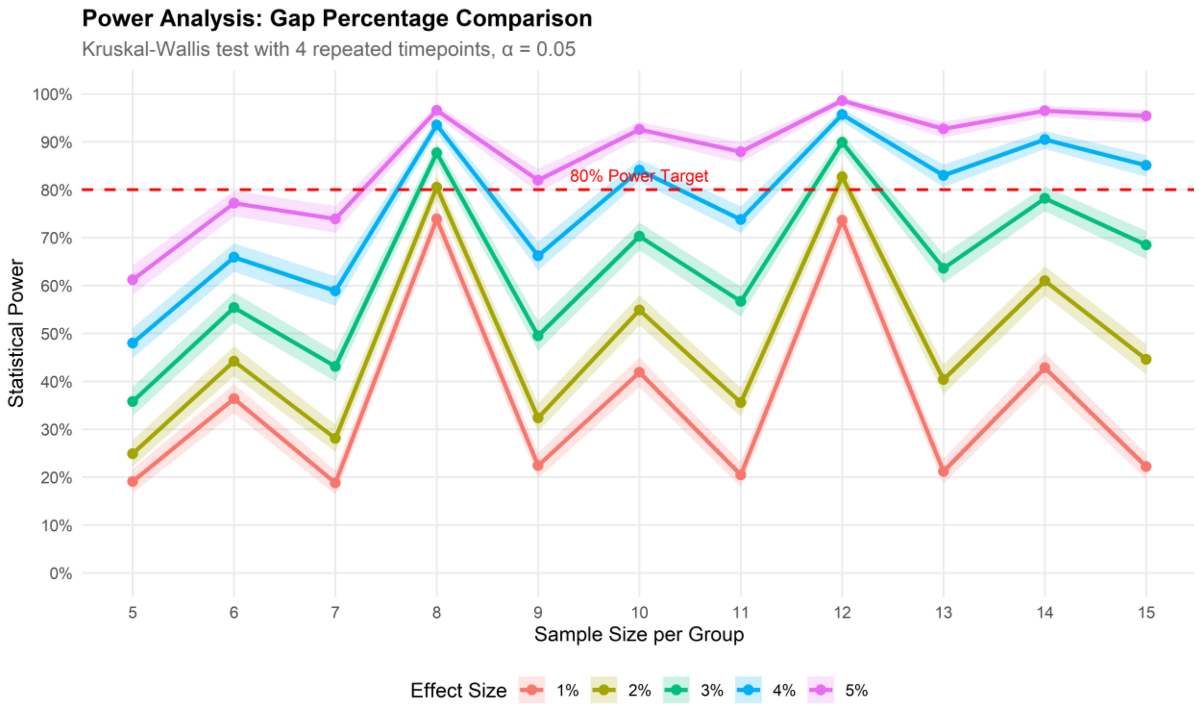

Figure S1: Power curves showing relationship between sample size and statistical power for different effect sizes

S5. Summary and Recommendations

```
all_effect_pcts <- sort(unique(results_df$effect_size_pct))

min_n_list <- lapply(all_effect_pcts, function(eff) {
  eff_data <- results_df[abs(results_df$effect_size_pct - eff) < 0.01, ]
  achieved <- eff_data[eff_data$power >= target_power, ]
  if (nrow(achieved) > 0) {
    min(achieved$n_per_group)
  } else {
    ">50"
  }
})
names(min_n_list) <- paste0(all_effect_pcts, "%")
```

S5.1 Key Findings

Based on 1000 simulations per condition using Kruskal-Wallis tests on beta-distributed gap percentage data with within-subject correlation of 0.6:

Minimum sample sizes to achieve 80% power:

- **1.0% effect size:** >50 samples per group
- **2.0% effect size:** 8 samples per group
- **3.0% effect size:** 8 samples per group
- **4.0% effect size:** 8 samples per group
- **5.0% effect size:** 8 samples per group

S5.2 Methodological Notes

1. **Data Generation:** Gap percentages were simulated using a beta distribution (appropriate for bounded 0-100% data) with mean ~10% for Group A and mean + effect size for Group B.
2. **Correlation Structure:** Within-subject correlation across timepoints was modeled using a multivariate normal copula approach, with correlation coefficient of 0.6.
3. **Statistical Test:** Kruskal-Wallis test was applied separately at each timepoint. Power was calculated as the proportion of simulations where at least one timepoint showed a significant difference ($p < 0.05$).

Confidence Intervals: 95% CIs for power estimates were calculated using exact binomial

Supplement Table S13. Materials, devices and software used in the study

**Category**	**Material / Device**	**Brand name**	**Manufacturer (City, Country)**	**LOT / Serial No.**	**Purpose**
Etchant	Phosphoric acid 37%	Total Etch	Ivoclar Vivadent (Schaan, Liechtenstein)	Z00NST	Enamel and dentin etching
Adhesive	Universal adhesive	Adhese® Universal VivaPen	Ivoclar Vivadent (Schaan, Liechtenstein)	Z00SFL	Tooth surface conditioning
Etchant	Ceramic etching gel	IPS® Ceramic Etching Gel	Ivoclar Vivadent (Schaan, Liechtenstein)		Ceramic surface conditioning
Primer	Universal ceramic primer	Monobond® Plus Universal Primer	Ivoclar Vivadent (Schaan, Liechtenstein)		Ceramic surface priming
Surface treatment	Al_2O_3(50 µm, 1,5 bar)	Basic quattro IS	Renfert (Hilzingen, Germany)	29580000	RBC conditioning
Luting agent	Dual-curing resin cement	Variolink® Esthetic DC neutral	Ivoclar Vivadent (Schaan, Liechtenstein)	Z00SBY	Adhesive luting
CAD/CAM material	Lithium disilicate ceramic	IPS e.max® CAD A3, HT	Ivoclar Vivadent (Schaan, Liechtenstein)	Z00F0C	Partial crowns (LS2)
CAD/CAM material	Resin-based composite	Tetric® CAD A3, HT	Ivoclar Vivadent (Schaan, Liechtenstein)	Z00R8G	Partial crowns (RBC)
Scanner	Intraoral scanner	CEREC Primescan	Dentsply Sirona (Bensheim, Germany)	-	Digital impression
Milling unit	CAD/CAM milling machine	CEREC MC XL	Dentsply Sirona (Bensheim, Germany)	-	Restoration milling
Imaging device	SD-OCT system	Telesto-II	Thorlabs (Dachau, Germany)	-	Interface imaging
